# Supplementary material for: Genetic Heterogeneity and Mutated PreS Analysis of Duck Hepatitis B Virus Recently Isolated from Ducks and Geese in China
Source: Animals (Basel). 2023 Apr 8;13(8):1282. doi: 10.3390/ani13081282 (PMC10135025; doi:10.3390/ani13081282)
Supplement: Supplementary file 1 [file animals-13-01282-s001.zip › animals-2270355-supplementary.pdf]

Supplementary Table S1: Detailed information regarding the stains used in this study.

Accession No.	Strain Name	Avian Species	Isolation Region	Genome Length
OQ183597	Y200825	Duck	Anhui, China	3024
OQ183598	Y200106	Duck	Henan, China	3024
OQ183599	Y201009	Duck	Hubei, China	3024
OQ183600	Y210928	Duck	Jiangsu, China	3027
OQ183601	Y220201	Duck	Henan, China	3027
OQ183602	Y211013	Duck	Anhui, China	3024
OQ183603	Y220217	Duck	Henan, China	3024
OQ183604	Y200122	Duck	Anhui, China	3027
OQ183605	Y200226	Duck	Henan, China	3024
OQ183606	Y200619	Duck	Anhui, China	3027
OQ183607	Y200722	Duck	Hubei, China	3024
OQ183608	Y210829	Duck	Jiangsu, China	3024
OQ183609	E200402	Goose	Anhui, China	3027
OQ183610	E200422	Goose	Hubei, China	3027
OQ183611	E200731	Goose	Henan, China	3027
OQ183612	E210501	Goose	Anhui, China	3027
OQ183613	E210321	Goose	Jiangsu, China	3027
OQ183614	E210526	Goose	Henan, China	3027
OQ183615	E210620	Goose	Hubei, China	3027
OQ183616	E210814	Goose	Jiangsu, China	3027
OQ183617	E220324	Goose	Hubei, China	3027
OQ183618	E200529	Goose	Anhui, China	3027
OQ183619	E200801	Goose	Hubei, China	3027
AF047045	DHBV-AF047045	Duck	Canada	3021
AF404406	DHBV-AF404406	Duck	China	3024
AF493986	Indiana	Duck	USA	3021
AF505512	DHBV-AF505512	Duck	USA	3021
AJ006350	DHBV-AJ006350	Duck	Australia	3027
AY250901	31	Duck	South Africa	3021
AY250902	102	Duck	South Africa	3021
AY250903	8	Duck	South Africa	3021
AY294028	ST10	Duck	South Africa	3021
AY294029	D1	Duck	South Africa	3021
AY294656	DHBV-AY294656	Duck	China	3021
AY392760	GD1	Duck	China	3027
AY433937	GD2	Duck	China	3027
AY521226	A	Duck	China	3027
AY521227	B	Duck	China	3027
AY536371	GD3	Duck	China	3027
DQ195079	3	Duck	Germany	3021
DQ276978	DHBV-DQ276978	Duck	China	3024
EU429324	CH4	Duck	China	3006

EU429325	CH5	Duck	China	3006
EU429326	CH6	Duck	China	3006
M21953	DHBV-M21953	Duck	China	3024
M32990	DHBV-M32990	Duck	China	3027
M32991	DHBV-M32991	Duck	China	3027
X12798	DHBV-X12798	Duck	Germany	3021
X58569	DHBV-X58569	Duck	Germany	3024
X74623	DHBV-X74623	Duck	India	3021
HQ214130	DHBV-XY	Duck	China	3021
MF471769	SD-02	Duck	China	3021
JX469896	DHBV	Duck	China	3027
AF110996	SGHBV1-13	Snow goose	Germany	3024
